# Reproductive isolation, evolutionary distinctiveness and setting conservation priorities: The case of European lake whitefish and the endangered North Sea houting (*Coregonus *spp.)

**DOI:** 10.1186/1471-2148-8-137

**Published:** 2008-05-09

**Authors:** Michael M Hansen, Dylan J Fraser, Thomas D Als, Karen-Lise D Mensberg

**Affiliations:** 1 Technical University of Denmark, National Institute of Aquatic Resources, Vejlsøvej 39, DK-8600 Silkeborg, Denmark; 2 Department of Biology, Dalhousie University, Halifax, Nova Scotia B3H 1J1, Canada

## Abstract

**Background:**

Adaptive radiation within fishes of the *Coregonus lavaretus *complex has created numerous morphs, posing significant challenges for taxonomy and conservation priorities. The highly endangered North Sea houting (*C. oxyrhynchus*; abbreviated NSH) has been considered a separate species from European lake whitefish (*C. lavaretus*; abbreviated ELW) due to morphological divergence and adaptation to oceanic salinities. However, its evolutionary and taxonomic status is controversial. We analysed microsatellite DNA polymorphism in nine populations from the Jutland Peninsula and the Baltic Sea, representing NSH (three populations, two of which are reintroduced) and ELW (six populations). The objectives were to: 1) analyse postglacial recolonization of whitefish in the region; 2) assess the evolutionary distinctiveness of NSH, and 3) apply several approaches for defining conservation units towards setting conservation priorities for NSH.

**Results:**

Bayesian cluster analyses of genetic differentiation identified four major groups, corresponding to NSH and three groups of ELW (Western Jutland, Central Jutland, Baltic Sea). Estimates of historical migration rates indicated recolonization in a north-eastern direction, suggesting that all except the Baltic Sea population predominantly represent postglacial recolonization via the ancient Elbe River. Contemporary gene flow has not occurred between NSH and ELW, with a divergence time within the last 4,000 years suggested from coalescence methods. NSH showed interbreeding with ELW when brought into contact by stocking. Thus, reproductive isolation of NSH was not absolute, although possible interbreeding beyond the F1 level could not be resolved.

**Conclusion:**

Fishes of the *C. lavaretus *complex in the Jutland Peninsula originate from the same recolonization event. NSH has evolved recently and its species status may be questioned due to incomplete reproductive isolation from ELW, but it was shown to merit consideration as an independent conservation unit. Yet, application of several approaches for defining conservation units generated mixed outcomes regarding its conservation priority. Within the total species complex, it remains one among many recently evolved unique forms. Its uniqueness and high conservation priority is more evident at a local geographical scale, where conservation efforts will also benefit populations of a number of other endangered species.

## Background

Pleistocene glaciations and subsequent postglacial recolonisation events have profoundly affected biota within the Northern Hemisphere in terms of the distribution of genetic diversity and phylogeographical lineages [1-3]. Evolution did not, however, cease after the last glaciation. Thus, in several cases it has been difficult to disentangle population differentiation that can be ascribed to postglacial processes, i.e. within the past ca. 10,000 years, *versus *longer term evolution pre-dating the last glaciation [4-6].

This problem is particularly evident in freshwater fishes such as whitefish (*Coregonus *spp.) and threespine stickleback (*Gasterosteus aculeatus*), both of which show extensive phenotypic variability and comprise sympatrically and allopatrically divergent morphs [7-9]. Polymorphisms involving both benthic and limnetic forms and differences in lateral plate number have been extensively studied in sticklebacks, and it has been found that similar morphs have evolved repeatedly and independently, primarily representing postglacial divergence [10-12]. In *Coregonus*, "dwarf" vs. "normal" sized morphs and morphs with high vs. low gill raker number have attracted considerable interest, and the situation appears highly complex. In North America dwarf-normal forms and high-low gill raker forms in lake whitefish (*C. clupeaformis*) have in some cases been found to represent different phylogeographical lineages and in other cases postglacial divergence [9,13]. Moreover, for dwarf-normal systems parallel evolution has been suggested to involve the same loci [14,15]. In the closely related European lake whitefish (*C. lavaretus*), most gill raker morphs appear to represent postglacial divergence [16,17] and there are striking examples of other phenotypic forms that have evolved within the past few thousand years [8,18].

Cases like these pose significant challenges in the context of setting conservation priorities. Are similar phenotypes mono- or polyphyletic in origin? How much of the phenotypic diversity represents long-term evolutionary processes (e.g. different phylogeographical lineages) and how much represents more recent (e.g. postglacial) evolution? How should the outcomes of recent versus longer-term evolution be weighted in conservation priorities? Important components of these questions involve the estimation of long- and short-term adaptive divergence and reproductive isolation [19-22].

**Figure 1:**
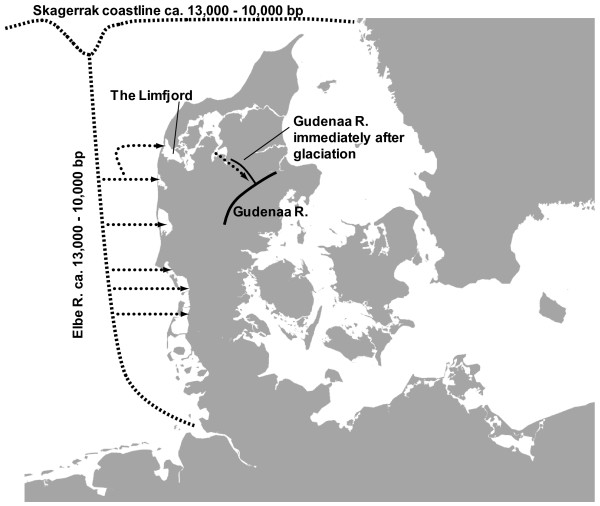
**Postglacial recolonisation route of *Coregonus *sp**. Map showing the most likely recolonisation routes of *Coregonus *sp. via the postglacial Elbe River system and further into the Limfjord. Immediately after the last glaciation the Gudenaa River flowed into the Limfjord, but later it changed outlet towards the Kattegat Sea [24]. Arrows indicate the direction of immigration.

The North Sea and Jutland peninsula region (Fig. 1) are interesting both in terms of geology and freshwater fish biogeography. Immediately after the last glaciation, ca. 13,000 years bp, the current North Sea, the Jutland Peninsula and the continent to the south was one coherent land mass. The postglacial Elbe River extended through this region and had its outlet in the current Skagerrak Sea, which was at that time a trough separating Norway and Sweden from Jutland. West flowing rivers in the Jutland Peninsula were tributaries to the postglacial Elbe River thus constituting one large river system. As glaciers melted, the sea level started to rise, and eventually the melting of the gigantic ice masses of the North American Wisconsinan glaciers from ca. 10,000 - 8,000 years bp led to the formation of the current North Sea, isolating the rivers of Western Jutland from the Elbe River system. Today, a number of freshwater fish species including grayling (*Thymallus thymallus*) and dace (*Leuciscus leuciscus*) are found only in rivers draining to the North Sea, presumably reflecting recolonization via the postglacial Elbe River system and absence of immigration via the Baltic Sea [23]. European lake whitefish (in the following denoted the *Coregonus lavaretus *complex, as this group of fishes involves several taxonomic controversies) shows a similar distribution pattern, although it spread further via ancient waterway connections into the Limfjord in northern Jutland and further to a few rivers, including the Gudenaa River on the Jutland East coast [24] (see Fig. 1).

The North Sea houting (hereafter denoted NSH), categorised by traditional taxonomy as *C. oxyrhynchus*, is a morphologically and ecologically divergent form of whitefish. Its most prominent feature is an elongated snout, which on average constitutes 6.2% of the total fork length (snout length is measured from the eye cavity to the tip of the snout). Conversely, in lake whitefish (hereafter denoted ELW) from the same geographical region the relative snout length is 4.6%, and the differences are statistically significant (Hvidt CB, Christensen IG: *Træk af Nordsøsnæblens (Coregonus oxyrhynchus L.) biologi i Vidå-systemet*. Aarhus, Denmark: Institute of Biology, University of Aarhus; 1990. M.Sc. Thesis). The feature of an elongated snout is nevertheless not unique to NSH, and has also been described in other populations [25]. However, what makes NSH stand out within the *C. lavaretus *complex is its ability to tolerate oceanic salinities [26]. Anadromy is not uncommon within this complex, involving migration into brackish environments such as fjords and the Baltic Sea with salinities ranging from near zero to approx. 15 ‰. In contrast, NSH undertakes feeding migrations into the North Sea, where salinity is approx. 35 ‰ [26].

NSH was previously distributed throughout the Wadden Sea area, a coastal zone of the North Sea extending from southern Jutland to the Netherlands characterized by huge tidal flats, where it spawned in tributary rivers, including major rivers like the Rhine and the Elbe. At present, all but one extant and indigenous population of NSH remains in the Danish Vidaa River, due to extensive pollution and habitat degradation. However, its species and conservation status is controversial. Based on microsatellite and mitochondrial DNA analysis Hansen *et al*. [24] found NSH to be closely related to other Danish ELW populations, but did not rule out that it represented a recent speciation event. Similarly, Østbye *et al*. [17] analysed mitochondrial DNA variation in populations of the *C. lavaretus *complex encompassing most of its distributional range in Europe and found that NSH did not constitute a divergent lineage relative to other populations. Finally, Freyhof & Schöter [27] analysed snout length and gill raker numbers in preserved specimens of whitefish from extant and extinct populations. They concluded that the remaining extant NSH population represents the same species as other Danish whitefish (named *C. maraena*), whereas the "true" NSH (i.e. *C. oxyrhynchus*) was restricted to the Rhine River region but is now extinct. However, the basis for this conclusion must be considered questionable, as homoplasy for morphological traits is common within *Coregonus *[9,16,17,28].

The extant NSH population is now subject to a large-scale rehabilitation programme, which involves habitat restoration and removal of impassable dams in rivers along the Danish Wadden Sea coast. NSH has also been reintroduced into other Danish and German rivers in the region. Nevertheless, knowledge of the evolutionary history of NSH and its relationships to ELW populations in the region remains scarce. The studies undertaken so far have either focused on the evolutionary history of the *C. lavaretus *complex on a large geographical scale [17] or made deductions about genetic relationships based on estimates of genetic differentiation [24]. Both in the context of setting conservation priorities and understanding the dynamics of postglacial phenotypic divergence there is an important need to obtain specific knowledge of historical and contemporary gene flow and to assess reproductive isolation between NSH and ELW.

**Figure 2:**
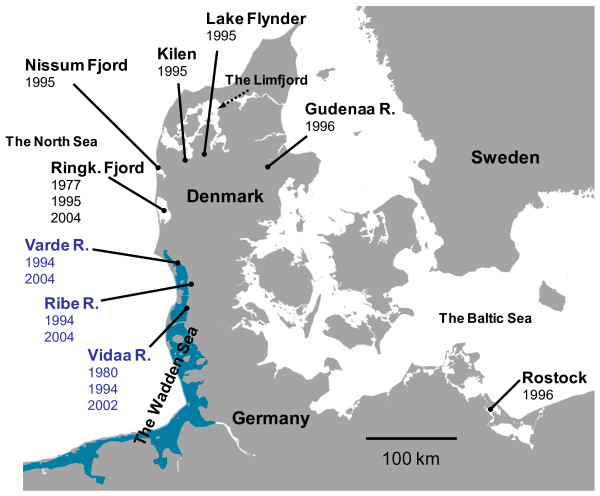
**Map showing sample localities**. Map showing the sampled localities and years of sampling. North Sea houting populations are denoted by blue print whereas European lake whitefish populations are written in black. The Wadden Sea is denoted by blue colour.

We analysed variation at 12 microsatellite DNA loci in 9 populations of the *C. lavaretus *complex (Table 1; Fig. 2). One ELW population was from the Baltic Sea, five populations represented ELW from the North Sea region, and NSH was represented by samples from the extant Vidaa River and samples from two rivers subject to reintroduction efforts. Temporal samples from 1977–1980 and to the present were available from the Vidaa (NSH) and the Ringkøbing Fjord (ELW) populations.

**Table 1:** Sample information

Population	Year of sampling	Sample code	Source of DNA	Taxonomy: ELW or NSH	Geographical region	Indigenous or reintroduced	Life history	Sample size
Vidaa River	2002	VID02	Adipose fin	NSH	Wadden (North) Sea	Indigenous	Anadromous (high salinity)	50
Vidaa River	1994	VID94	Adipose fin	NSH	Wadden (North) Sea	Indigenous	Anadromous (high salinity)	40
Vidaa River	1980	VID80	Dried scales	NSH	Wadden (North) Sea	Indigenous	Anadromous (high salinity)	39
Ribe River	2004	RIB04	Adipose fin	NSH	Wadden (North) Sea	Reintroduced	Anadromous (high salinity)	49
Ribe River	1994	RIB94	Adipose fin	NSH	Wadden (North) Sea	Reintroduced	Anadromous (high salinity)	29
Varde River	2004	VAR04	Adipose fin	NSH	Wadden (North) Sea	Reintroduced	Anadromous (high salinity)	36
Varde River	1994	VAR94	Adipose fin	NSH	Wadden (North) Sea	Reintroduced	Anadromous (high salinity)	19
Ringkøbing Fjord	2004	RIN04	Adipose fin	ELW	North Sea	Indigenous	Anadromous (brackish)	33
Ringkøbing Fjord	1995	RIN95	Adipose fin	ELW	North Sea	Indigenous	Anadromous (brackish)	50
Ringkøbing Fjord	1977	RIN77	Dried scales	ELW	North Sea	Indigenous	Anadromous (brackish)	37
Nissum Fjord	1995	NIS	Adipose fin	ELW	North Sea	Indigenous	Anadromous (brackish)	50
Kilen	1995	KIL	Adipose fin	ELW	The Limfjord	Indigenous	Lake	24
Lake Flynder	1995	FLY	Adipose fin	ELW	The Limfjord	Indigenous	Lake	40
Gudenaa River	1996	GUD	Adipose fin	ELW	Kattegat Sea	Indigenous	Anadromous (brackish)	35
Rostock (Achterwasser)	1996	ROS	Adipose fin	ELW	Baltic Sea	Indigenous	Anadromous (brackish)	34

Information about the sampled populations, year of sampling, sample codes, source of DNA, taxonomic status, geographical region of the sample localities, the indigenous or reintroduced status of populations and sample size. ELW denotes European lake whitefish (*Coregonus lavaretus*) and NSH North Sea Houting (*C. oxyrhynchus*).

The main objectives of the study were to analyze postglacial recolonization patterns and assess the evolutionary distinctiveness of NSH *vs*. ELW. This was achieved by analysing contemporary and historical gene flow and reproductive isolation between populations. Based on these results we assessed whether NSH fulfils the criteria of the Biological Species Concept [29], and we used the frameworks described by Waples [20], Moritz [30], Crandall *et al*. [21] and Green [31] for assessing its conservation priority.

## Methods

### Samples and populations

We analysed 15 samples from 9 populations, summarized in Table 1. All except two populations are anadromous, but only NSH populations migrate into a fully saline environment, whereas the other populations migrate into brackish environments (< 15 ‰). Two of the samples (VID80 and RIN77) consisted of archived scale samples, whereas the remaining samples consisted of adipose fin clips stored in 96% ethanol. The fish were caught either by electrofishing (samples from rivers) or by net (samples from fjords and lakes).

### Microsatellite DNA analysis

DNA was extracted from tissue samples using either phenol-chloform extraction [32] or proteinase K – Chelex extraction [33]. DNA from archived scale samples were extracted using a phenol-chloform extraction procedure modified for "ancient" DNA [34]. Twelve microsatellite loci were analysed: *Sfo23 *[35], *Bwf1 *and *Bwf2 *[36], *BFRO018 *[37], *C2-157 *[38], *Cocl-Lav1*, *Cocl-Lav4*, *Cocl-Lav6*, *Cocl-Lav18*, *Cocl-Lav27*, *Cocl-Lav49 *and *Cocl-Lav52 *[39]. Details about PCR conditions, electrophoresis and scoring are given in [40].

### Statistical analyses

Deviations from Hardy-Weinberg equilibrium were tested by exact tests [41] using GENEPOP 3.1d [42]. Genetic variation within samples was quantified by observed (*H*_*o*_) and expected (*H*_*e*_) heterozygosity, and by allelic richness [43], a measure of the number of alleles independent of sample size.

We tested for outlier status and possible selection at each of the microsatellite loci using the method and software FDIST2 [44]. We assumed a model of 50 populations exchanging migrants and sample sizes of 30 individuals and further based the simulations on a step-wise mutation model.

Genetic differentiation was estimated by *θ*_*ST *_[45] and by *R*_*ST*_, the latter of which incorporates mutational information and assumes a step-wise mutation model [46]. The software ARLEQUIN 3.0 [47] was used for estimating *θ*_*ST *_and the significance was tested by permuting individuals 100,000 times among samples. *R*_*ST *_was estimated using SPAGEDI 1.1 [48], and by permuting allele sizes among alleles (10,000 times) it was tested whether *R*_*ST *_was significantly higher than *θ*_*ST *_[49]. The genetic relationships among populations were further visualized by a multidimensional scaling plot based on *θ*_*ST *_between pairs of samples, using VISTA 5.6.3 (Young FW. ViSta: The Visual Statistics System. *Research Memorandum 94-1(b) (2nd. ed.)*. 1996. Chapel Hill, NC, L.L.Thursone Psychometric Laboratory, University of North Carolina). All tests for significance of Hardy-Weinberg disequilibrium and pairwise *θ*_*ST *_and *R*_*ST *_were corrected for multiple tests by calculating the False Dicovery Rate (FDR) [50].

We used the Bayesian clustering approach implemented in STRUCTURE 2.2 [51,52] for 1) estimating the number of populations/groups represented by the sampled individuals (*k*) and 2) assigning individuals to populations without using prior information of the sample of origin. For estimating the most likely *k *we conducted runs assuming *k *= 1..10. We assumed an admixture model and correlated allele frequencies. Each run consisted of a burn-in of 100,000 Markov Chain Monte Carlo (MCMC) steps, followed by 500,000 steps. Ten replicates were conducted for each *k*. We plotted the probability of the data [(P(D)] and the *ad hoc *statistic *Δk *[53], the latter of which measures the steepest increase of the probability of *k*. STRUCTURE 2.2 was furthermore used for estimating individual admixture proportions in the VAR94 and VAR04 samples, which represent an introduced NSH population that we suspected had become admixed with ELW. We used individuals from VID and RIN as baseline samples, chose the option "use popinfo" for VID and RIN individuals, assumed an admixture model and correlated allele frequencies, and then estimated individual admixture proportions and their 90% posterior probability intervals for the VAR94 and VAR04 individuals.

Population level admixture proportions in VAR94 and VAR04 were analysed using the method and software ADMIX 2.0 [54], with standard deviations estimated based on bootstrapping 10,000 times over loci. Given that admixture was expected to have occurred recently, we did not consider molecular distances between alleles.

Contemporary gene flow among most populations was considered unlikely, as this would require migration through highly saline water. However, given that NSH can tolerate oceanic salinities, gene flow may occur between VID, RIB and VAR and the neighbouring RIN and NIS ELW populations. This was analysed using the individual assignment based method BAYESASS 1.3 [55], which estimates migration rates based on the inferred proportion of immigrants within the past two generations. Initial runs showed that genetic differentiation was too low for estimating migration involving VID and RIB and RIN and NIS, respectively, and due to possible hybridization in VAR (see Results) this population was not included. Consequently, VID and RIB and RIN and NIS were pooled into two groups of populations. Three independent runs showed that 10^7 ^MCMC steps, of which the first 10^6 ^steps were burn-in, were sufficient to achieve convergence. We then conducted a final analysis based on 2 × 10^7 ^MCMC steps, of which 2 × 10^6 ^steps were burn-in.

We further estimated effective population size (*N*_*e*_) in VID and RIN using 1) a temporal method which assumes a closed population [56], and 2) a temporal method which assumes a population open to gene flow and estimates immigration rate (*m*) and *N*_*e *_[57]. This was based on the temporal samples from VID taken in 1980, 1994 and 2002, and from RIN taken in 1977, 1995 and 2004. When *N*_*e *_and *m *was estimated for VID, all temporal samples from RIN were pooled to represent a possible source of migrants and *vice versa*. We assumed a generation length of 4 years for NSH [40] and for RIN ELW we estimated the mean age of reproduction to be 2.92 (~3) years based on unpublished data from scale readings, and used this value as a proxy for generation length.

Historical gene flow and effective population size was analysed as implemented in the software MIGRATE 2.0.3 [58]. The method is based on a coalescence model with mutation and migration and estimates a measure of effective population size, *θ*, defined as 4*N*_*e*_*μ*, where *μ *denotes mutation rate, and migration *M*, defined as *m/μ*, where *m *denotes migration rate. To provide unscaled parameters we estimated mutation rates for the individual loci based on an analysis of the GUD sample using the method MSVAR 2.0 [59]. This approach yielded mutation rates ranging from 1.01 × 10^-4 ^to 9.00 × 10^-4 ^for individual loci, with a geometric mean value of 2.81 × 10^-4^. This value corresponds well with an estimate of mutation rate for microsatellite loci in *Cyprinus carpio *of 5.56 × 10^-4 ^[60] and a mean mutation rate of ca. 10^-4 ^for microsatellites in humans [61]. We assumed a step-wise mutation model and based estimates on fifteen short (10^4 ^MCMC steps) and five long (10^5 ^steps) chains. To ensure convergence, we used the "adaptive heating" option with one "cold" and three "hot" chains. Three runs were conducted, with run number two and three using the estimates of the previous run as starting parameters. The results did not change much after the first analysis, suggesting that convergence had been achieved, and we report the results of the third run. We omitted the RIB and VAR populations from the analyses, as they are assumed to represent introduced populations, and we used the most recent samples from the VID and RIN populations (VID02 and RIN04).

Finally, we estimated historical effective population sizes, migration rates and splitting time between the VID and RIN populations using the Bayesian, coalescence and MCMC based method IMa [62]. The method assumes an isolation-with-gene-flow model, where a single population at some point back in time splits into two, which are subsequently connected by some level of gene flow. The following parameters are estimated: *θ*_*A*_, the effective population size in the ancestral population prior to splitting; *θ*_1 _and *θ*_2_, the effective population size of population 1 and 2, respectively, after splitting; *m*_1 _and *m*_2_, the migration rate from population 2 into population 1 and *vice versa*; and *t*, the point back in time when the ancestral population split into two. All parameters are scaled by mutation rate. To provide unscaled parameters we used the same mutation rate estimate as described for MIGRATE (see above). We conducted several initial runs and found that a heated chain approach with 25 chains (parameters g1 = 0.0013 and g2 = 2) and an initial burn-in of 10^6 ^MCMC steps followed by 4 × 10^6 ^steps was required to reach convergence. We assumed a generation time of four years and the following additional parameters: q1 = q2 = qA = 40, m1 = m2 = 40 and t = 10.

## Results

### Genetic variation and relationships among populations

Variation at the 12 microsatellite loci ranged from 4 to 34 alleles (see Additional file 1). Eleven significant deviations from Hardy-Weinberg equilibrium were observed among a total of 180 tests (6.1%). Three deviations were observed at the locus *Sfo23 *and three at locus *Cocl-Lav1*. However, there were no general patterns suggesting null alleles at these loci, although we cannot rule out that null alleles may be present at varying frequencies among populations.

*θ*_*ST *_[45] among all populations was moderate but statistically highly significant (0.069, p < 0.0001; temporal samples from the same populations were pooled). A test for outlier status of individual loci using FDIST2 [44] identified one locus, *Cocl-Lav27*, as a significant outlier (*θ*_*ST *_= 0.249, p < 0.0001). This locus showed low variation (a total of four alleles; see Additional file 1), and one allele (183 bp) exhibited a frequency of 0.857 in VID, whereas another allele (185 bp) was present at a frequency of 0.838 in ROS. We were unable to distinguish if diversifying selection is acting on this locus or if the strong differentiation is due to a combination of a signal of postglacial recolonization and low variability. However, we omitted the locus from analyses based on coalescence (MIGRATE and IMa), where selection at a locus could result in seriously misleading outcomes.

Pairwise *θ*_*ST *_between all samples showed low and mainly non-significant differentiation between temporal samples from the NSH population VID and the ELW population RIN, whereas significant differentiation was observed between temporal samples of NSH from RIB and VAR (Table 2). Significant differentiation was evident between samples of NSH from VID and RIB *vs*. the two geographically most proximate ELW populations, RIN and NIS (*θ*_*ST *_ranging between 0.055 and 0.108). Curiously, however, the VAR sample from 2004 showed closer genetic relationships to the RIN and NIS ELW samples (*θ*_*ST *_from 0.017 – 0.033) compared to the VAR sample from 1994. The GUD ELW population showed relatively close relationships to RIN and NIS and more distant relationships to the other populations. Both Limfjord populations (KIL, FLY) and particularly the Baltic Sea population ROS, showed strong genetic differentiation to other populations (pairwise *θ*_*ST *_up to 0.184).

**Table 2:** *θ*_*ST *_and *R*_*ST *_between pairs of samples

	VID02	VID94	VID80	RIB04	RIB94	VAR04	VAR94	RIN04	RIN95	RIN77	NIS	KIL	FLY	GUD	ROS
**VID02**		0.002	0.000	0.011***	0.040***	0.027***	0.021***	0.065***	0.083***	0.080***	0.067***	0.128***	0.130***	0.066***	0.166***
**VID94**	0.008		0.003	0.015***	0.025***	0.031***	0.022***	0.078***	0.100***	0.095***	0.078***	0.141***	0.148***	0.076***	0.182***
**VID80**	-0.002	0.007		0.007	0.024***	0.023***	0.011	0.055***	0.072***	0.069***	0.056***	0.128***	0.126***	0.050***	0.161***
**RIB04**	-0.001	-0.003	0.001		0.020***	0.029***	0.008	0.066***	0.080***	0.082***	0.070***	0.127***	0.118***	0.065***	0.161***
**RIB94**	0.005	0.000	0.012	-0.003		0.041***	0.024***	0.083***	0.108***	0.108***	0.094***	0.149***	0.144***	0.086***	0.184***
**VAR04**	0.026*	0.016	0.017	0.024*	0.032*		0.034***	0.017***	0.032***	0.033***	0.028***	0.074***	0.078***	0.027***	0.114***
**VAR94**	-0.002	0.002	0.013	-0.003	-0.006	0.000		0.066***	0.069***	0.079***	0.056***	0.129***	0.140***	0.056***	0.171***
RIN04	0.072***	0.082***	0.046*	0.081***	0.104***	0.029	0.064*		0.007	0.006	0.006	0.055***	0.069***	0.015***	0.079***
RIN95	0.055***	0.040**	0.040**	0.057***	0.069***	0.010	0.034	0.008		0.011**	0.012***	0.066***	0.072***	0.021***	0.088***
RIN77	0.099***	0.095***	0.075***	0.098***	0.122***	0.025	0.072*	0.000	-0.002		0.012**	0.064***	0.070***	0.025***	0.094***
NIS	0.124***	0.088***	0.096***	0.109***	0.122***	0.031*	0.080**	0.052**	0.018	0.025*		0.063***	0.086***	0.017***	0.085***
KIL	0.094***	0.123**	0.080*	0.105**	0.087*	0.103**	0.077	0.087*	0.140***	0.087*	0.190***		0.059***	0.077***	0.093***
FLY	0.037**	0.061***	0.028	0.044**	0.042*	0.033*	0.022	0.053**	0.077***	0.059**	0.108***	0.034		0.093***	0.125***
GUD	0.022**	0.032**	0.010	0.027*	0.041*	0.001	0.012	0.002	0.011	0.001	0.045**	0.079*	0.018		0.094***
ROS	0.119***	0.094***	0.079***	0.099***	0.093***	0.060***	0.084***	0.078***	0.081***	0.082***	0.062***	0.097**	0.064***	0.065***	

*θ*_*ST *_[45] (above diagonal) and *R*_*ST *_[46] (below diagonal) between all pairs of samples, along with tests of their significance. Samples of North Sea houting are denoted by boldface. * significant at the 5% level, ** significant at the 1% level, *** significant at the 0.1% level

**Figure 3:**
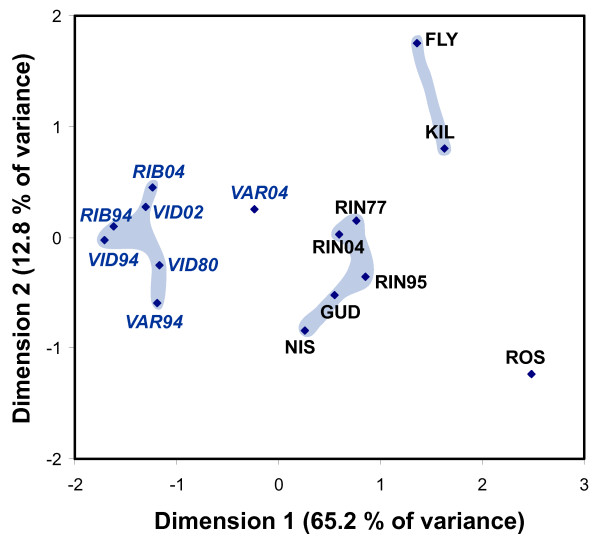
**Multidimensional scaling plot of genetic relationships among samples**. Multidimensional scaling plot based on pairwise *θ*_*ST *_between samples. Samples of North Sea houting are denoted by blue print. See Table 1 for a list of sample abbreviations.

A multidimensional scaling plot based on pairwise *θ*_*ST *_values identified four groups of samples separated along dimensions 1 and 2 (Fig. 3). The first consisted of NSH samples from VID and RIB along with the VAR sample from 1994. The second consisted of ELW samples from RIN, NIS and GUD. The VAR sample from 2004 was intermediate between these two groups. The two Limfjord populations KIL and FLY formed a third group, whereas the fourth group consisted only of ROS, which was distinct from all other populations. Applying other genetic distances, such as Nei's D_A _[63] yielded essentially similar results.

*R*_*ST *_was 0.036 (p < 0.001) among all populations. Pairwise *R*_*ST *_values between samples are shown in Table 2. If *R*_*ST *_> *F*_*ST *_(or *θ*_*ST*_) this would imply that mutation as opposed to drift plays a significant role in creating genetic differentiation among populations, suggesting that divergence has occurred over very long time scales. However, the test for *R*_*ST *_> *θ*_*ST *_[49] yielded no evidence for a higher *R*_*ST *_than *θ*_*ST *_among all samples. Tests between pairs of populations, after pooling temporal samples within populations, also yielded no significant outcomes across all loci (results not shown). At the single locus level, 22 tests among a total of 432 tests yielded p-values < 0.05 (without correction for multiple tests). Five of these involved the locus *Sfo23 *and the ROS population, which was the most divergent population in terms of *θ*_*ST *_(see Table 2), whereas thirteen tests involved the locus *bwf1 *and samples of North Sea houting from VID, RIB and VAR. Thus, we do not entirely rule out that mutation has played a minor role in generating genetic divergence, but drift has played the predominant role.

### Bayesian clustering of individuals

**Figure 4:**
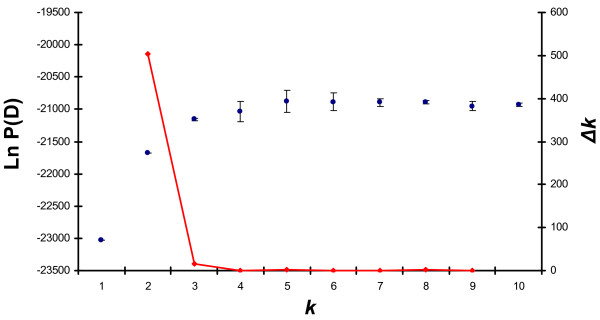
**Probability of number of clusters represented by the data**. Probability of the data set representing 1..10 clusters [P(D)], as determined by replicate analyses using STRUCTURE 2.2 [51,52] (black points +/- s.d.), and the *ad hoc *statistic *Δk*, measuring the steepness of increase of P(D) [53] (red line).

**Figure 5:**
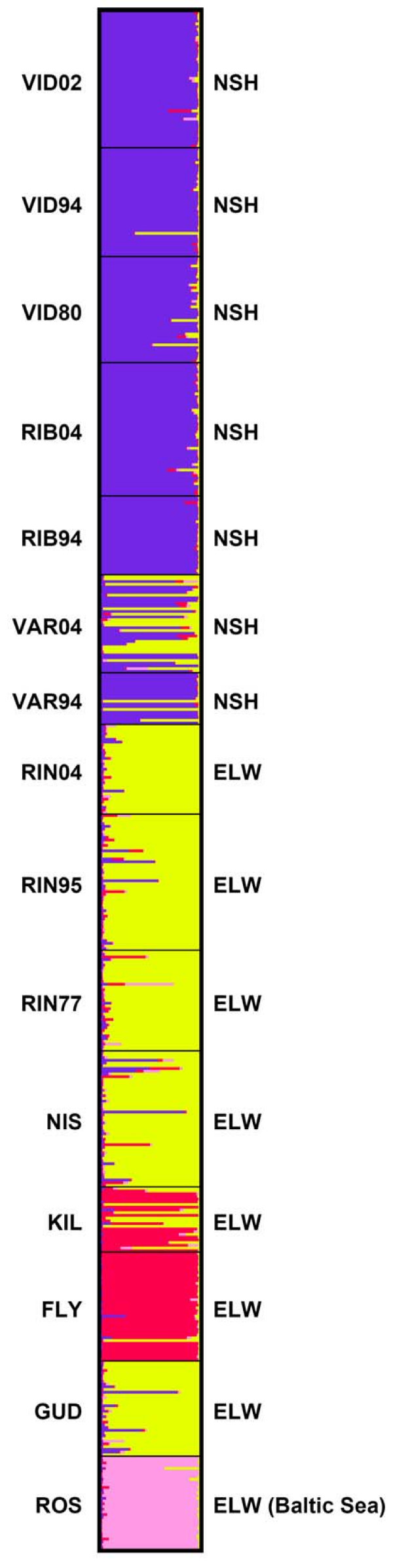
**Bayesian clustering of individuals**. Bayesian clustering of all individuals using STRUCTURE 2.2 [51,52], assuming four different clusters of individuals (*k *= 4), an admixture model and no prior population information. Each horizontal bar denotes an individual, and the four colours denote the different inferred clusters. NSH denotes North Sea houting, whereas ELW denotes European lake whitefish populations. See Table 1 for a list of sample abbreviations.

Replicate estimation of the probability of the data [P(D)] using STRUCTURE 2.2 [51,52] and assuming *k *= 1..10 showed that P(D) reached a plateau for *k *= 4 (Fig. 4). Conversely, estimation of *Δk*, which measures the steepest increase in P(D) [53], was clearly highest for *k *= 2 (Fig. 4). Inspection of the partition of individuals showed that individuals from NSH samples were split from the remaining populations at *k *= 2, but *k *= 3 and 4 provided further partitioning of populations. Assuming *k *> 4 did not identify more groups, and we therefore conclude that *k *= 4 captures most of the biological information in the data. The partitioning of individuals at *k *= 4 is shown in Fig. 5. The first cluster consists of individuals from NSH populations (VID and RIB), and the second cluster consists of individuals from the ELW populations RIN, NIS and GUD. Interestingly, the VAR94 and particularly VAR04 samples appear to consist of a mixture of these two groups. This analysis, involving all samples and not making use of learning samples, does not resolve whether or not interbreeding occurs between NSH and ELW. Consequently, we conducted more detailed analyses of individual admixture proportions in VAR using VID and RIN as baseline samples and assuming *k *= 2 (see below). The third cluster consists of the populations FLY and partly KIL, both from the Limfjord region, whereas the ROS population from the Baltic Sea makes up the fourth cluster. Analyses of the four clusters separately did not identify additional groups (data not shown).

### Contemporary gene flow and effective population size

Contemporary gene flow estimated using BAYESASS 1.3 [55] was very low: 0.0055 (95% CI 0.0001 – 0.0167) from NSH to ELW (i.e. VID/RIB to RIN/NIS) and 0.0049 (95% CI 0.0002 – 0.0162) from ELW to NSH (i.e. RIN/NIS to VID/RIB).

Effective population size (*N*_*e*_) estimated using a temporal method assuming no migration [56] yielded values of 522 and 521, respectively, in VID and RIN (Table 3). Using a method which assumes a population open to gene flow and which simultaneously estimates *N*_*e *_and *m *(immigration rate) [57] yielded very low *m *values, i.e. 0.0007 in VID and 0.0062 in RIN. However, under this assumption *N*_*e *_estimates differed considerably; 1139 in VID and 273 in RIN (Table 3), demonstrating that *N*_*e *_estimates with this method are highly sensitive towards inferred migration rates [64].

**Table 3:** Effective population size and migration rate

Focal population	Model assuming gene flow	*N*_*e *_(95% CI)	*m *(95% CI)
VID	No	522.3 (213.7 – >4000)	NA
	Yes	1139.3 (1137.3 – 1141.3)	0.0007 (< 0.0001 – 0.0048)
RIN	No	521.4 (264.1 – 2641.2)	NA
	Yes	272.9 (160.7 – 527.9)	0.0062 (0.0050 – 0.0120)

Estimates of effective population size (*N*_*e*_) and migration rate (*m*) and their 95% confidence intervals in VID and RIN, using two temporal methods assuming a closed population [56] and a population open to gene flow [57], respectively.

### Historical gene flow and splitting time

**Figure 6:**
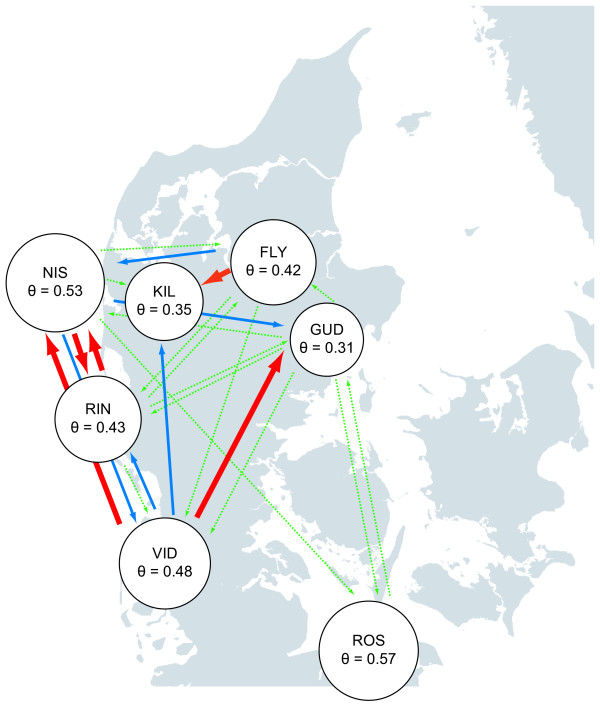
**Historical effective population sizes and migration rates**. Map with superimposed historical effective population size estimates (*θ*) of the sampled indigenous *Coregonus *populations. Gene flow estimates (*M*) and the direction of gene flow are indicated by arrows. Punctuated green arrows indicate 10 = *M *< 15, blue arrows indicate 15 = *M *< 20 and red arrows indicate *M *= 20. Estimates of *M *< 10 are not shown. See Additional file 2 for an overview of all *θ *and *M *values and their associated 95% confidence intervals. The analyses were conducted using the method and software MIGRATE 2.0.3 [58].

Analysis of historical demographic parameters using MIGRATE 2.0.3 [58] yielded *θ *values ranging between 0.31 (GUD) and 0.57 (ROS) (Fig. 6). Assuming a geometric mean mutation rate of 2.81 × 10^-4 ^as estimated for the loci analysed in this study, this would correspond to historical *N*_*e *_estimates ranging from 276 to 508. Historical migration rate estimates, *M*, ranged from 4.8 to 36.4 (see Fig. 6 and Additional file 2). Assuming the same mutation rate as above this corresponds to migration rates ranging between 0.001 and 0.010. The direction of migration and approximate migration rates are shown in Fig. 6. The results suggest historical migration in a north-eastern direction from VID, along the Jutland North Sea coast and via the Limfjord, with bidirectional gene flow occuring within regions (i.e. VID, RIN, NIS and FLY, KIL). ROS from the Baltic Sea was the most isolated population.

Estimation of splitting time between the indigenous NSH population VID and the geographically most proximate ELW population RIN using the IMa software [62] resulted in a scaled splitting time parameter, *t*, of 0.174 (Table 4). Assuming the estimated mutation rate of 2.81 × 10^-4 ^this corresponds to 2482 years before present (90% credible interval 641 – 4344 years). The inferred estimates of historical *N*_*e *_and *m *showed good correspondence with those obtained using MIGRATE (Table 4).

**Table 4:** Splitting time, historical effective population size and gene flow

Scaled parameters					
*θ*_1_	*θ*_2_	*θ*_*A*_	*m*_1_	*m*_2_	*t*
0.395 (0.392 – 3.532)	2.040 (1.510–7.550)	40.637 (20.638–59.900)	4.758 (0.020 – 11.620)	16.791 (0.020–29.020)	0.174 (0.045–0.305)

Unscaled parameters, assuming μ = 2.81 × 10^-4^					
*N*_1_	*N*_2_	*N*_*A*_	*m*_1_	*m*_2_	*t*

352 (349 – 3143)	1816 (1344 – 6720)	36170 (18369 – 53316)	0.001 (0.000 – 0.003)	0.005 (0.000 – 0.008)	2482 (641 – 4344)

Estimates of historical demographic parameters and their 90% credible intervals in VID and RIN, analysed using IMa [62] and assuming that these two populations have split from a common ancestral population. Parameters scaled by mutation rate and unscaled parameters are provided. Scaled parameters: *θ*_1_, *θ*_2_, *θ*_*A *_= historical effective population size in VID, RIN and the ancestral population, respectively, *m*_1_, *m*_2 _= migration rate from RIN to VID and VID to RIN, respectively, and *t *= splitting time. Unscaled parameters: *N*_1_, *N*_2_, *N*_*A *_= historical effective population size (individuals) in VID, RIN and the ancestral population, respectively, *m*_1_, *m*_2 _= migration rate (individuals per generation) from RIN to VID and VID to RIN, respectively, and *t *= splitting time (years).

### Admixture analyses

Several of the analyses suggested that the VAR population, which was supposed to represent NSH reintroduced from VID, was in fact admixed with ELW. We obtained information that the VAR river had been stocked with ELW in the 1980s originating from either KIL or RIN (Peter Geertz-Hansen, Technical University of Denmark, National Institute of Aquatic Resources, personal communication). However, the results using STRUCTURE 2.2 (Fig. 5) pointed to RIN or NIS as the most likely contributors rather than KIL. Analysis of admixture proportions using ADMIX 2.0 [54] and assuming VID, RIN and KIL as baseline populations showed a negative admixture proportion of KIL in the VAR94 sample, suggesting that KIL had not contributed to admixture, whereas VID and RIN contributed approx. 80 and 20%, respectively (Table 5). A positive, albeit small admixture proportion of KIL (9%), was found in the VAR04 sample, whereas VID contributed 50% and RIN 41% (Table 5). Thus, we conclude that VAR is an admixed population with the major proportions contributed by VID and RIN, whereas KIL has made a minor, if any contribution.

**Table 5:** Admixture proportions

Admixed sample		Baseline samples	
	VID	RIN	KIL
	
VAR94	0.833 (s.d. 0.082)	0.205 (s.d.0.155)	-0.041 (s.d. 0.098)
VAR04	0.501 (s.d. 0.057)	0.412 (s.d. 0.084)	0.088 (s.d. 0.059)

Admixture proportions in samples of putative NSH from VAR, taken in 1994 and 2004. Baseline samples consisted of NSH from VID and ELW from RIN and KIL. The analyses were conducted using the method and software ADMIX 2.0 [54].

**Figure 7:**
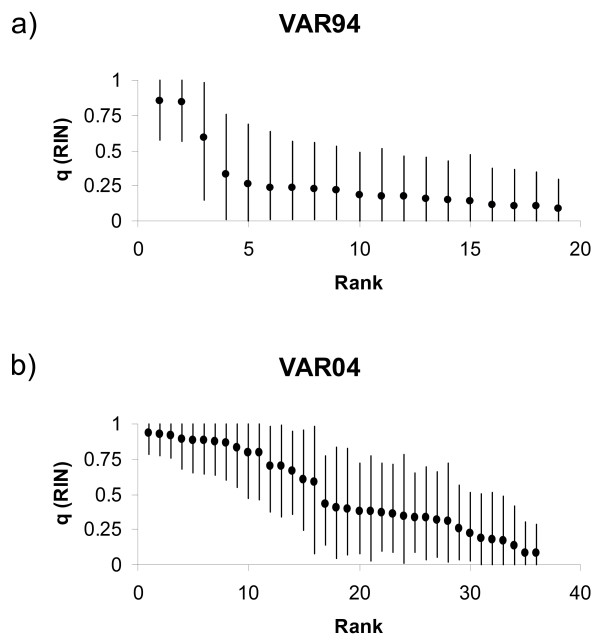
**Individual admixture proportions**. Individual admixture proportions (*q*) and their associated 90% credible intervals in the VAR94 and VAR04 samples, estimated using VID (NSH) and RIN (ELW) as learning samples. A *q *value of 1 denotes a "pure" RIN individual, and conversely 0 denotes a "pure" VID individual. *q *values were ranked from lowest to highest. The analyses were conducted using STRUCTURE 2.2 [51,52]. a) *q *values in the VAR94 sample. b) *q *values in the VAR04 sample.

We further used STRUCTURE 2.2 [51,52] for analysing individual admixture proportions (*q*) in VAR94 and VAR04 using VID and RIN as learning samples. In the VAR94 sample there was limited evidence for hybridization, although two individuals showed intermediate *q *values (Fig. 7a). The VAR04 sample, however, contained several individuals showing intermediate *q *values and with 90% confidence intervals not including 0 and 1 (Fig. 7b). A total of 16 individuals (44%) showed individual admixture proportions of RIN ranging from 0.25 to 0.75. We cannot be certain that they all represent admixed individuals and the results do not allow for distinguishing F1 hybrids from backcrosses. Nevertheless, the results provide evidence that NSH and ELW are not reproductively isolated when they occur in sympatry.

## Discussion

Our study's results suggest that the sampled populations constitute four major groups, one of which represents NSH, and that all populations except ROS from the Baltic Sea show modest genetic differentiation, presumably reflecting a recent common ancestry and a single postglacial recolonization event. Moreover, the two geographically closest indigenous populations of NSH and ELW (VID and RIN) must be considered reproductively isolated at present but have nevertheless split recently, perhaps within the past few thousand years. Finally, NSH and ELW appear to interbreed where they have been brought into contact in one locality (VAR). We discuss these findings below along with their implications for species designation and for prioritizing populations for conservation.

### Postglacial recolonization and genetic population structure

Subfossil remains of fish from the *Coregonus lavaretus *complex, northern pike (*Esox lucius*), burbot (*Lota lota*), ruffe (*Acerina cernua*) and smelt (*Osmerus operlanus*) have all been recovered from a lake deposit in northern Jutland and dated to approximately 11,000 years bp [65]. Hence, whitefish were among the first freshwater fish species to recolonize the Jutland Peninsula. In general, whitefishes, northern pike and burbot exhibit a circumpolar distribution and bear genetic signatures of rapid postglacial expansion [17,66-69], thereby conforming to the definition of pioneer species *sensu *Hewitt [70]. There was moderate genetic differentiation among all samples (excluding ROS) and the tests for *R*_*ST *_> *θ*_*ST *_[49] suggested that mutation has played a minor role in generating population differentiation. This indeed suggests rapid recolonization of the Jutland Peninsula, predominantly from a single source. The inferred direction of historical gene flow (Fig. 6) also supports a recolonization event from the south to north via the postglacial Elbe River system and further into the Limfjord, though some historical migration may also have occurred in the opposite direction within regions. This result is in accordance with the study by Østbye *et al*. [17] based on mitochondrial DNA sequence data, which suggested a northward expansion of a Central European lineage, encompassing the Jutland Peninsula. However, this study also found a minor contribution of a separate Northern European lineage in several Danish populations, a finding that would be difficult to confirm by analyzing microsatellite DNA markers. Moreover, our results do not allow for distinguishing whether this admixture of lineages occurred prior to postglacial recolonization, or if a separate minor recolonization event by the Northern European lineage took place.

The Bayesian clustering of individuals (Fig. 5) might at first seem to contradict a common source of recolonization of all populations, given that ELW from RIN, NIS and GUD formed one cluster, whereas the two populations from the Limfjord, KIL and FLY, formed a separate cluster. These latter two populations are interspersed between NIS and GUD on the supposed route of recolonization via the Elbe River (Fig. 1), so it seems illogical that they should not belong to the same cluster. However, whereas the environment in GUD has been relatively stable in the long term, the Limfjord is a highly dynamic region, which has experienced repeated opening and closure of the connection to the North Sea. The latest disconnection from the North Sea lasted from ca. 1100 to 1825, and during that period the main part of the Limfjord was brackish and supported large populations of ELW [71]. In 1825, a storm reconnected the Limfjord and the North Sea, and the increased salinity caused immediate extirpation of most whitefish. The FLY and KIL populations can be considered remnants of these large populations, and we suggest that repeated extinctions, recolonizations and population size fluctuations during cycles of brackish and high salinity conditions have led to stronger drift and changes in allele frequencies as compared to other Danish ELW populations.

Whereas gene flow has occurred historically between populations, they must now be considered reproductively isolated, although contemporary gene flow might still take place between certain ELW populations owing to their low genetic differentiation (e.g. RIN and NIS). The region inhabited by RIN and NIS is marshy with several smaller lakes and rivers, which could become connected at high water levels. Hence, occasional gene flow is certainly a possibility. Available temporal *N*_*e *_estimates (under the assumption of no gene flow) in RIN and VID, were also relatively high (point estimates exceeded 500). Detailed analyses of the demographic history of VID further showed that *N*_*e *_has been stable over the past ca. 20 years [40]. We cannot be certain that current *N*_*e *_is also high in the other samples for which temporal samples were not available. However, since historical *N*_*e *_(*θ*) did not vary much among populations (Fig. 6), it is reasonable to assume that all populations are relatively large. Hence, NSH and ELW of the Jutland Peninsula can be described as a system of relatively large populations sharing a common postglacial ancestry, which are now reproductively isolated and potentially following separate evolutionary trajectories.

### Reproductive isolation and species designation

The taxonomy of the *Coregonus lavaretus *complex is chaotic and numerous species have been described [72]. These species designations have been based on the Phylogenetic Species Concept [73], which defines species as the smallest lineages or population groups that can be united by *synapomorphic *characters, i.e. shared characters derived from a common ancestor. NSH has traditionally been considered a species (*C. oxyrhynchus*) separate from ELW (*C. lavaretus*) based particularly on its elongated snout and its ability to tolerate oceanic salinities [26]. It has also sometimes been denoted as a high gill raker morph, although by comparison to other *C. lavaretus *populations the gill raker numbers must be considered intermediate; the mean gill raker number is 32.1 (s.d. 1.7) in VID *vs*. a range of mean values from 16.8 – 46.0 in other European populations, and a range from 29.1 to 35.5 in RIN, NIS, FLY and GUD (see supplementary material in [17]). Hence, gill raker number does not separate NSH from geographically proximate ELW populations. On the other hand, NSH is distinct by showing a higher age at reproduction than ELW; 4.0 years in NSH from VID vs. 2.9 years in ELW from RIN ([40]; this study), and a considerably higher mean length at reproduction in VID than in RIN (ca. 45 cm vs. 35 cm; Hvidt CB, Christensen IG: *Træk af Nordsøsnæblens (Coregonus oxyrhynchus L.) biologi i Vidå-systemet*. Aarhus, Denmark: Institute of Biology, University of Aarhus; 1990. M.Sc. Thesis). All together, these characters might justify separate species designation of NSH adopting a Phylogenetic Species Concept, but the lack of demonstration of the genetic basis of these characters and absence of analysis of phenotype-environment associations would inevitably cast doubt on this classification.

From an evolutionary biology perspective, adoption of the Biological Species Concept [29] is more satisfactory, as it directly addresses the issue of reproductive isolation; species are defined as biological entities that show reproductive isolation even when they occur in sympatry. The presence of both ELW and NSH in VAR provides a rare opportunity for testing reproductive isolation in sympatry, and the results suggest interbreeding in the VAR04 sample, as 16 out of 36 individuals showed individual admixture coefficients ranging between 0.25 and 0.75 (Fig. 7b). It is puzzling that there is a lower contribution of ELW and fewer hybrids in the VAR94 sample. Based on records from 1994 we assume that this is due to biased sampling, where individuals with ELW morphology were omitted.

The results suggest that NSH and ELW may not qualify as separate species according to a Biological Species Concept. The acid test would involve demonstration of interbreeding beyond the F1 generation and lack of postzygotic selection against hybrids, which could otherwise act to maintain species integrity despite interbreeding. There was insufficient statistical power in the STRUCTURE analyses to unambiguously identify backcrosses, which would require analysis of many more loci given the relatively low genetic differentiation [74]. It should be noted that further analyses using the method NEWHYBRIDS [75] yielded qualitatively similar results, but also failed to distinguish F1 hybrids from F2 and backcrosses (data not shown).

A more pragmatic alternative to the Biological Species Concept would be Mallet's concept of Genotypic Clusters [76], where species are identified as groups of individuals separated into discrete clusters of genetic variation. This concept allows for interbreeding between species as long as clustering is maintained. NSH and ELW have recently come into contact in VAR and some discreteness of clustering would be expected even in the case of random mating. However, continuous monitoring of individual clustering using STRUCTURE could provide a test of species integrity according to the concept of Genotypic Clusters.

Although NSH and ELW do not show reproductive isolation in VAR, their sympatric occurrence is an artefact resulting from stocking. Different statistical methods based on fundamentally different principles [55,57,62] all suggest contemporary reproductive isolation between allopatric NSH and ELW populations. However, estimated splitting time between VID and RIN was surprisingly recent, ranging between 600 – 4300 years under the assumption of the estimated mutation rate of 2.81 × 10^-4 ^(Table 4). The formation of the Wadden Sea dates back to the rise of sea level 8,000 – 10,000 years bp, though it has since then been a dynamic region [77]. The estimate of splitting time is highly dependent on the assumed mutation rate, and it would require only a slightly lower mutation rate, 1.5 × 10^-4^, for the 90% credible interval for splitting time to encompass 8,000 years. We therefore conclude that NSH and ELW have split within the past few thousand years, but we are unable to provide a more exact timing. Given that NSH inhabits the Wadden Sea area we find it most plausible that its divergence is associated with the formation of this marine region.

The question remains whether the VID population is in fact a remnant of a single species or morph previously distributed throughout the Wadden Sea region. Coregonids show extensive potential for homoplasy in morphological traits [9,16,28], and the possibility certainly exists that similar morphs have evolved repeatedly and independently within the region from the Rhine River in the South to the Varde River in the North. Answering this question would require analysis of samples from other, now extinct populations. Analysis of DNA from archived historical samples, such as scales, is probably the only option for resolving this issue [34].

### Conservation priorities

Questioning species designation can be contentious, as species have previously been conceived as the primary units for conservation. However, it is becoming increasingly apparent that conservation should also recognize intraspecific diversity to conserve the evolutionary legacy of species [20-22]. In the following we apply four different approaches for defining conservation units to the case of NSH vs. ELW.

The Evolutionarily Significant Unit (ESU) concept as defined by Waples [20] states that an ESU is a population or group of populations that shows *substantial reproductive isolation *from other populations and constitutes *an important component of the evolutionary legacy of the species*. Under natural circumstances NSH does show reproductive isolation from ELW populations in the region. However, most allopatric ELW would also fulfill that criterion. NSH is also morphologically distinct and shows adaptation to oceanic salinities. The latter property could be regarded as an important component of the evolutionary legacy of the *C. lavaretus *complex, though it ranks as one among a number of other recently evolved remarkable features found among populations within the complex (see e.g. [8,18]). Hence, NSH would qualify as an ESU, but so would numerous other recently diverged forms. This would be biologically defendable [78], but would lead to a very large number of units with limited resources to protect them all. On the scale of the North Sea region and the Jutland Peninsula, NSH would stand out more clearly as a distinct unit.

The second approach by Moritz [30] adopts the criteria that ESUs should exhibit *reciprocal monophyly for mtDNA haplotypes *and *significant genetic differentiation at nuclear loci*. This approach operates with a second category, management units (MU), i.e. populations showing significant differentiation at nuclear loci but not reciprocal monophyly at mtDNA. As this ESU definition stresses the time-frame over which populations have been reproductively isolated, it is unsurprising that NSH does not qualify as an ESU; mtDNA haplotypes in NSH vs. neighbouring ELW populations do not show reciprocal monophyly [17]. Instead, the three phylogeographical lineages identified by Østbye *et al*. [17] would be designated as ESUs. Given the significant differentiation at microsatellite loci, NSH would be categorized as an MU within the Central European lineage, but most of the other studied populations would also be granted MU status.

Crandall *et al.'s *[21] framework for defining conservation units assesses both reproductive isolation and adaptive divergence by considering hypotheses of *genetic exchangeability *(reproductive isolation) and *ecological exchangeability *(essentially adaptive divergence). Moreover, these criteria are considered on both a contemporary and historical time scale. We reject the hypothesis of contemporary genetic exchangeability between all populations. If we define historical exchangeability as dating back to postglacial recolonization, then we accept the hypothesis of historical genetic exchangeability of all populations. However, if "historical" is defined as pre-dating major anthropogenic influence, tentatively 500 years bp, then we reject the hypothesis for most populations, including VID vs. RIN.

The data on ecological exchangeability is much scarcer. We are not aware of any adaptive divergence among the ELW populations of the study. This, however, does not preclude that adaptive divergence actually exists. The only feature that really stands out is high salinity tolerance in VID (NSH) and we therefore reject the hypothesis of contemporary ecological exchangeability between NSH and ELW. There are no long-term data relating to ecological exchangeability. If we consider a postglacial time-scale, then we would assume that ecological exchangeability existed, while it is most likely that high salinity tolerance was present in VID (NSH) if we consider a time scale of 500 years bp. Depending on the definition of historical exchangeability the recommendation would then be to treat VID (NSH) as a distinct species (500 years time scale) or a distinct population (postglacial time scale).

Finally, the Designatable Unit (DU) described by Green [31] is closely related to Waples' ESU definition [20], but additionally takes extinction risk into account. As previously discussed, NSH must be considered a separate unit, though its uniqueness depends on the scale considered. It is virtually impossible to assess extinction risk across the whole *C. lavaretus *complex, but on a local scale none of the studied ELW populations are considered endangered. The VID population appears stable and not immediately endangered [40] but is nevertheless the only remaining indigenous population of NSH. Hence, NSH and the VID population would qualify as a Designatable Unit, but its conservation priority and need for a recovery plan would require more detailed analysis of extinction risk.

Altogether, NSH stands out as a separate conservation unit, although its distinctiveness varies considerably depending on the framework applied. Moreover, prioritization depends on the spatial or temporal scale considered, with highest prioritization granted on finer or shorter scales.

The current rehabilitation program for NSH is based on restoring habitat in four rivers in the Danish region of the Wadden Sea, including the Vidaa River. These rivers harbour a number of other endangered species listed in the Berne Convention on the Conservation of European Wildlife and Natural Habitats. This includes the last remnants of indigenous Atlantic salmon (*Salmo salar*) populations in the region from the Jutland Peninsula to Northern France [79], twaite shad (*Alosa fallax*) and sea lamprey (*Petromyzon marinus*), all of which would benefit from a NSH conservation program. Thus, an integrated approach combining conservation priority of NSH relative to other *C. lavaretus *forms with principles for identifying areas of importance for biodiversity conservation [80] might be a fruitful way to settle uncertainties, and would undoubtedly result in higher priority for the rehabilitation program as a whole.

## Conclusion

We conclude that fishes of the *C. lavaretus *complex that have recolonized the Jutland Peninsula after the last glaciation are the result of recolonization, which mainly or exclusively took place via the ancient Elbe River system. The present populations arose due to vicariance following rising sea levels that disconnected water bodies from the Elbe River.

Based on all existing scientific information, NSH is genetically and ecologically distinct albeit closely related to ELW populations in the same region. In particular, the ability of NSH to tolerate high salinities is unique within the *C. lavaretus *complex. Estimates of splitting time suggest that divergence has occurred within the past few thousand years. Natural gene flow does not occur at present between NSH and ELW, but the observation of interbreeding in one location where they have been brought into contact by stocking, suggests that reproductive isolation is incomplete. Depending on the degree of interbreeding beyond the F1 level and the presence or absence of postzygotic reproductive barriers this may call the separate species status of NSH and ELW into question, and NSH is perhaps best regarded as an ecologically and morphologically divergent morph and incipient species. NSH should be managed separately from ELW populations in the region, but the degree to which it should be prioritized depends on the spatial or temporal scale considered.

The case of the North Sea houting and European lake whitefish provides an important example of postglacially evolved biodiversity in the Northern Hemisphere. It also illustrates the challenges for setting conservation priorities for species complexes exhibiting a shallow phylogeny but potentially considerable phenotypic and adaptive diversity. Even though analysis of molecular markers may greatly increase knowledge about genetic contingency and reproductive isolation, evidence for adaptive divergence in most cases remains scarce and circumstantial, unless demanding tests such as common garden experiments are conducted. There are also no ideal frameworks available for assessing conservation priorities at the subspecific level. Even putatively objective methods involve subjectivity, such as defining time scales for historical exchangeability using Crandall *et al*.'s [21] approach or defining the geographical scales to consider. This calls for pragmatism, where the subjectivity involved is acknowledged and clearly presented. Finally, just as identification of conservation units should consider intraspecific biodiversity, our study shows that consideration of biodiversity at the species level in the region of interest may help to settle otherwise unclear cases of conservation priorities.

## Authors' contributions

MMH conceived and planned the study, conducted the statistical analyses and had the leading role in writing the paper. DJF assisted with the interpretation of results, contributed expertise on principles for defining management units and contributed to the writing of the paper. TDA assisted with the interpretation of results, contributed to the writing of the paper and prepared most of the figures. K–LDM screened and optimized the molecular markers and conducted all the laboratory work. All authors read and approved the final manuscript.

## Supplementary Material

Additional file 1 Summary data per locus and sample. Summary statistics including estimates of genetic variation and tests for Hardy-Weinberg equilibrium at each locus in each sample. Click here for file

Additional file 2 Historical effective population size and migration rates. Estimates of historical effective population size (*θ*) and migration rate between populations (*M*), estimated using MIGRATE 2.0.3 [58]. Click here for file
