# Supplementary material for: Reproductive isolation, evolutionary distinctiveness and setting conservation priorities: The case of European lake whitefish and the endangered North Sea houting (Coregonus spp.)
Source: BMC Evol Biol. 2008 May 9;8:137. doi: 10.1186/1471-2148-8-137 (PMC2396634; doi:10.1186/1471-2148-8-137)
Supplement: Additional file 1 — Summary data per locus and sample. Summary statistics including estimates of genetic variation and tests for Hardy-Weinberg equilibrium at each locus in each sample. [file 1471-2148-8-137-S1.doc]

Supplementary Table 1. Summary of total number of observed alleles per locus, allele size ranges for the loci, allelic richness (AR) based on the smallest sample size in the data set, i.e. n = 14 for locus *Cocl-Lav4* in VAR94, outcome of tests for deviations from expected Hardy-Weinberg proportions (H.-W. test), expected (H_e_) and observed heterozygosity (H_o_), and sample sizes (N) of the studied populations.

| Locus |  | VID02 | VID94 | VID80 | RIB04 | RIB94 | VAR04 | VAR94 | RIN04 |
| --- | --- | --- | --- | --- | --- | --- | --- | --- | --- |
| *Sfo23* | Allelic richness | 8.4 | 8.5 | 8.5 | 8.7 | 8.9 | 12.2 | 5.9 | 14.8 |
| Total no. | H.-W. test | 0.0647 | 0.0289 | 0.1408 | 0.0064^*^ | 0.0104^*^ | 0.0381 | 0.2631 | 0.0257 |
| alleles: 34 | H_o_ | 0.840 | 0.925 | 0.846 | 0.878 | 0.966 | 0.778 | 0.684 | 0.909 |
| Size range: | H_e_ | 0.823 | 0.824 | 0.849 | 0.883 | 0.871 | 0.899 | 0.735 | 0.941 |
| 147-257 | N | 50 | 40 | 39 | 49 | 29 | 36 | 19 | 33 |
| *BWF2* | Allelic richness | 4.9 | 5.7 | 5.6 | 5.1 | 5.7 | 7.2 | 4.9 | 5.5 |
| Total no. | H.-W. test | 0.2082 | 0.5396 | 0.3440 | 0.7773 | 0.1460 | 0.0397 | 0.6889 | 0.0146 |
| alleles: 9 | H_o_ | 0.700 | 0.775 | 0.763 | 0.735 | 0.828 | 0.743 | 0.737 | 0.697 |
| Size range: | H_e_ | 0.734 | 0.785 | 0.746 | 0.749 | 0.785 | 0.822 | 0.698 | 0.760 |
| 148-164 | N | 50 | 40 | 38 | 49 | 29 | 34 | 19 | 33 |
| *BWF1* | Allelic richness | 6.4 | 5.9 | 7.0 | 6.9 | 6.5 | 7.3 | 7.3 | 6.5 |
| Total no. | H.-W. test | 0.4985 | 0.9383 | 0.1693 | 0.0806 | 0.2664 | 0.0246 | 0.6316 | 0.6188 |
| alleles: 12 | H_o_ | 0.760 | 0.675 | 0.718 | 0.633 | 0.679 | 0.514 | 0.647 | 0.697 |
| Size range: | H_e_ | 0.750 | 0.705 | 0.781 | 0.818 | 0.779 | 0.733 | 0.736 | 0.749 |
| 211-233 | N | 50 | 40 | 39 | 49 | 29 | 36 | 17 | 33 |

| Locus |  | VID02 | VID94 | VID80 | RIB04 | RIB94 | VAR04 | VAR94 | RIN04 |
| --- | --- | --- | --- | --- | --- | --- | --- | --- | --- |
| *Cocl-Lav1* | Allelic richness | 3.0 | 3.7 | 3.1 | 3.6 | 1.9 | 3.0 | 3.0 | 3.8 |
| Total no. | H.-W. test | 0.1582 | 0.0004^**^ | 0.4914 | 0.4694 | 0.0526 | 0.0000^***^ | 0.0004^**^ | 0.3630 |
| alleles: 4 | H_o_ | 0.660 | 0.400 | 0.462 | 0.531 | 0.034 | 0.306 | 0.211 | 0.576 |
| Size range: | H_e_ | 0.583 | 0.572 | 0.547 | 0.576 | 0.100 | 0.542 | 0.576 | 0.614 |
| 239-251 | N | 50 | 40 | 39 | 49 | 29 | 36 | 19 | 33 |
| *BFRO018* | Allelic richness | 4.1 | 3.9 | 3.9 | 4.5 | 4.1 | 4.0 | 3.7 | 3.3 |
| Total no. | H.-W. test | 0.3270 | 0.2973 | 0.8479 | 0.3153 | 0.0292 | 0.4052 | 0.8252 | 0.0897 |
| alleles: 7 | H_o_ | 0.640 | 0.625 | 0.692 | 0.551 | 0.690 | 0.500 | 0.579 | 0.333 |
| Size range: | H_e_ | 0.640 | 0.620 | 0.654 | 0.607 | 0.595 | 0.554 | 0.677 | 0.467 |
| 188-202 | N | 50 | 40 | 39 | 49 | 29 | 36 | 19 | 33 |
| *C2-157* | Allelic richness | 5.4 | 6.0 | 5.7 | 5.9 | 6.2 | 7.8 | 7.9 | 3.8 |
| Total no. | H.-W. test | 0.5038 | 0.0355 | 0.2824 | 0.7685 | 0.0800 | 0.5826 | 0.0165 | 0.1189 |
| alleles: 20 | H_o_ | 0.740 | 0.564 | 0.605 | 0.735 | 0.821 | 0.771 | 0.579 | 0.636 |
| Size range: | H_e_ | 0.742 | 0.729 | 0.775 | 0.757 | 0.799 | 0.791 | 0.812 | 0.656 |
| 120-172 | N | 50 | 39 | 38 | 49 | 28 | 36 | 19 | 33 |
| *Cocl-Lav4* | Allelic richness | 3.0 | 2.6 | 3.0 | 2.5 | 2.7 | 5.0 | 2.0 | 4.7 |
| Total no. | H.-W. test | 1.0000 | 1.0000 | 0.1701 | 1.0000 | 0.3356 | 0.0447 | 1.0000 | 0.6485 |
| alleles: 11 | H_o_ | 0.300 | 0.200 | 0.211 | 0.143 | 0.172 | 0.417 | 0.143 | 0.697 |
| Size range: | H_e_ | 0.267 | 0.187 | 0.220 | 0.137 | 0.222 | 0.482 | 0.138 | 0.565 |
| 142-168 | N | 50 | 39 | 39 | 49 | 29 | 36 | 14 | 33 |
| Locus |  | VID02 | VID94 | VID80 | RIB04 | RIB94 | VAR04 | VAR94 | RIN04 |
| *Cocl-Lav6* | Allelic richness | 6.1 | 5.5 | 6.6 | 6.1 | 5.8 | 8.3 | 7.9 | 8.9 |
| Total no. | H.-W. test | 0.0325 | 0.1121 | 0.8510 | 0.9146 | 0.7527 | 0.1574 | 0.5102 | 0.6609 |
| alleles: 18 | H_o_ | 0.600 | 0.825 | 0.846 | 0.714 | 0.724 | 0.833 | 0.789 | 0.906 |
| Size range: | H_e_ | 0.693 | 0.737 | 0.760 | 0.671 | 0.728 | 0.842 | 0.782 | 0.881 |
| 121-191 | N | 50 | 40 | 39 | 49 | 29 | 36 | 19 | 33 |
| *Cocl-Lav18* | Allelic richness | 3.0 | 3.0 | 3.0 | 3.0 | 3.0 | 2.9 | 3.0 | 3.4 |
| Total no. | H.-W. test | 0.4356 | 0.1588 | 0.8288 | 0.3217 | 0.7214 | 1.0000 | 0.0256 | 0.3741 |
| alleles: 5 | H_o_ | 0.500 | 0.475 | 0.590 | 0.612 | 0.690 | 0.361 | 0.316 | 0.333 |
| Size range: | H_e_ | 0.458 | 0.497 | 0.512 | 0.508 | 0.606 | 0.336 | 0.434 | 0.386 |
| 148-158 | N | 50 | 40 | 39 | 49 | 29 | 36 | 19 | 32 |
| *Cocl-Lav27* | Allelic richness | 2.5 | 2.0 | 2.3 | 2.0 | 2.0 | 2.4 | 2.7 | 2.9 |
| Total no. | H.-W. test | 0.4454 | 0.3963 | 1.0000 | 1.0000 | 1.0000 | 0.0211 | 1.0000 | 0.0664 |
| alleles: 4 | H_o_ | 0.280 | 0.175 | 0.205 | 0.224 | 0.069 | 0.278 | 0.158 | 0.485 |
| Size range: | H_e_ | 0.331 | 0.202 | 0.189 | 0.201 | 0.068 | 0.450 | 0.152 | 0.576 |
| 181-187 | N | 50 | 40 | 39 | 49 | 29 | 36 | 19 | 33 |
| *Cocl-Lav49* | Allelic richness | 7.0 | 7.8 | 7.6 | 8.0 | 8.2 | 10.5 | 6.8 | 8.2 |
| Total no. | H.-W. test | 0.9820 | 0.6356 | 0.9780 | 0.1969 | 0.8371 | 0.6536 | 0.2176 | 0.4337 |
| alleles: 15 | H_o_ | 0.900 | 0.850 | 0.892 | 0.857 | 0.893 | 0.917 | 0.765 | 0.788 |
| Size range: | H_e_ | 0.831 | 0.853 | 0.865 | 0.863 | 0.858 | 0.893 | 0.854 | 0.818 |
| 167-199 | N | 50 | 40 | 37 | 49 | 27 | 36 | 18 | 33 |

| Locus |  | VID02 | VID94 | VID80 | RIB04 | RIB94 | VAR04 | VAR94 | RIN04 |
| --- | --- | --- | --- | --- | --- | --- | --- | --- | --- |
| *Cocl-Lav52* | Allelic richness | 9.0 | 8.7 | 11.1 | 9.5 | 8.3 | 13.3 | 9.5 | 13.0 |
| Total no. | H.-W. test | 0.9583 | 0.2665 | 0.3065 | 0.7896 | 0.7258 | 0.0039^*^ | 0.1238 | 0.4516 |
| alleles: 31 | H_o_ | 0.980 | 1.000 | 0.974 | 1.000 | 1.000 | 1.000 | 1.000 | 0.970 |
| Size range: | H_e_ | 0.878 | 0.866 | 0.897 | 0.888 | 0.847 | 0.919 | 0.869 | 0.922 |
| 101-169 | N | 49 | 40 | 39 | 49 | 27 | 36 | 16 | 33 |

| Locus |  | RIN94 | RIN77 | NIS | FLY | KIL | GUD | ROS |
| --- | --- | --- | --- | --- | --- | --- | --- | --- |
| *Sfo23* | Allelic richness | 13.2 | 15.6 | 12.9 | 9.6 | 9.7 | 9.9 | 16.2 |
|  | H.-W. test | 0.1064 | 0.0580 | 0.2156 | 0.1225 | 0.0011^**^ | 0.0385 | 0.9219 |
|  | H_o_ | 0.880 | 0.917 | 0.860 | 0.900 | 0.667 | 0.857 | 0.939 |
|  | H_e_ | 0.900 | 0.949 | 0.920 | 0.852 | 0.817 | 0.875 | 0.943 |
|  | N | 50 | 37 | 50 | 40 | 24 | 35 | 34 |
| *BWF1* | Allelic richness | 6.0 | 5.7 | 5.2 | 4.5 | 5.6 | 4.9 | 5.5 |
|  | H.-W. test | 0.1295 | 0.0543 | 0.6049 | 0.1761 | 0.7538 | 0.2474 | 0.9929 |
|  | H_o_ | 0.800 | 0.595 | 0.660 | 0.625 | 0.583 | 0.686 | 0.706 |
|  | H_e_ | 0.758 | 0.649 | 0.632 | 0.597 | 0.676 | 0.703 | 0.683 |
|  | N | 50 | 36 | 50 | 40 | 24 | 35 | 33 |
| *BWF2* | Allelic richness | 5.4 | 6.8 | 5.8 | 7.0 | 7.4 | 4.4 | 6.1 |
|  | H.-W. test | 0.4153 | 0.1177 | 0.6659 | 0.0001^***^ | 0.0036 | 0.7029 | 0.8453 |
|  | H_o_ | 0.760 | 0.919 | 0.720 | 0.850 | 0.696 | 0.629 | 0.706 |
|  | H_e_ | 0.723 | 0.779 | 0.705 | 0.833 | 0.833 | 0.705 | 0.718 |
|  | N | 50 | 37 | 50 | 40 | 23 | 35 | 34 |

| Locus |  | RIN94 | RIN77 | NIS | FLY | KIL | GUD | ROS |
| --- | --- | --- | --- | --- | --- | --- | --- | --- |
| *BFRO018* | Allelic richness | 3.3 | 3.4 | 3.3 | 3.0 | 3.0 | 3.0 | 3.0 |
|  | H.-W. test | 0.2728 | 0.1331 | 0.3449 | 0.4598 | 0.0054 | 0.0990 | 0.0017^**^ |
|  | H_o_ | 0.540 | 0.568 | 0.560 | 0.675 | 0.333 | 0.429 | 0.324 |
|  | H_e_ | 0.654 | 0.677 | 0.605 | 0.626 | 0.616 | 0.604 | 0.557 |
|  | N | 50 | 37 | 50 | 40 | 24 | 35 | 34 |
| *Cocl-Lav1* | Allelic richness | 2.6 | 3.4 | 3.1 | 2.3 | 2.0 | 4.0 | 2.7 |
|  | H.-W. test | 1.0000 | 0.8550 | 0.0447 | 0.3208 | 0.2351 | 0.8218 | 0.3422 |
|  | H_o_ | 0.286 | 0.378 | 0.400 | 0.150 | 0.263 | 0.600 | 0.303 |
|  | H_e_ | 0.282 | 0.396 | 0.527 | 0.184 | 0.371 | 0.617 | 0.290 |
|  | N | 49 | 37 | 50 | 40 | 19 | 35 | 33 |
| *Cocl-Lav4* | Allelic richness | 6.0 | 5.0 | 6.7 | 6.4 | 9.7 | 7.4 | 6.4 |
|  | H.-W. test | 0.0499 | 0.9277 | 0.7559 | 0.4296 | 0.0203 | 0.7497 | 0.3153 |
|  | H_o_ | 0.660 | 0.676 | 0.740 | 0.675 | 0.708 | 0.857 | 0.794 |
|  | H_e_ | 0.728 | 0.669 | 0.767 | 0.721 | 0.804 | 0.757 | 0.811 |
|  | N | 50 | 37 | 50 | 40 | 24 | 35 | 34 |
| *Cocl-Lav6* | Allelic richness | 4.2 | 4.5 | 5.7 | 3.3 | 4.8 | 4.1 | 6.2 |
|  | H.-W. test | 0.5685 | 0.0766 | 0.1566 | 0.0025^**^ | 0.3980 | 0.8543 | 0.6819 |
|  | H_o_ | 0.440 | 0.486 | 0.500 | 0.500 | 0.708 | 0.400 | 0.765 |
|  | H_e_ | 0.446 | 0.505 | 0.560 | 0.483 | 0.675 | 0.373 | 0.769 |
|  | N | 50 | 37 | 50 | 40 | 24 | 35 | 34 |
| Locus |  | RIN94 | RIN77 | NIS | FLY | KIL | GUD | ROS |
| *Cocl-Lav18* | Allelic richness | 9.6 | 8.9 | 7.4 | 3.9 | 8.4 | 9.4 | 6.6 |
|  | H.-W. test | 0.7783 | 0.2406 | 0.4896 | 0.0102^*^ | 0.2896 | 0.1716 | 0.1210 |
|  | H_o_ | 0.900 | 0.848 | 0.820 | 0.500 | 0.826 | 0.857 | 0.706 |
|  | H_e_ | 0.872 | 0.873 | 0.828 | 0.685 | 0.862 | 0.878 | 0.788 |
|  | N | 50 | 37 | 50 | 40 | 24 | 35 | 34 |
| *Cocl-Lav27* | Allelic richness | 2.9 | 2.9 | 3.0 | 3.7 | 2.8 | 3.0 | 2.9 |
|  | H.-W. test | 0.2577 | 1.0000 | 0.6450 | 0.7135 | 0.3983 | 0.4686 | 0.1907 |
|  | H_o_ | 0.300 | 0.351 | 0.500 | 0.375 | 0.208 | 0.514 | 0.706 |
|  | H_e_ | 0.329 | 0.308 | 0.447 | 0.368 | 0.263 | 0.469 | 0.540 |
|  | N | 50 | 33 | 50 | 40 | 23 | 35 | 34 |
| *Cocl-Lav49* | Allelic richness | 2.9 | 2.9 | 3.0 | 3.9 | 3.0 | 2.9 | 3.1 |
|  | H.-W. test | 0.1010 | 1.0000 | 1.0000 | 0.1433 | 0.5049 | 0.0355 | 0.6178 |
|  | H_o_ | 0.440 | 0.556 | 0.600 | 0.625 | 0.417 | 0.629 | 0.265 |
|  | H_e_ | 0.568 | 0.557 | 0.584 | 0.637 | 0.462 | 0.535 | 0.287 |
|  | N | 50 | 36 | 50 | 40 | 24 | 35 | 34 |
| *Cocl-Lav52* | Allelic richness | 8.0 | 8.7 | 9.9 | 4.6 | 6.9 | 8.6 | 7.7 |
|  | H.-W. test | 0.7049 | 0.9358 | 0.2201 | 0.1400 | 0.5161 | 0.7300 | 0.5309 |
|  | H_o_ | 0.820 | 0.857 | 0.920 | 0.725 | 0.696 | 0.800 | 0.794 |
|  | H_e_ | 0.823 | 0.848 | 0.877 | 0.683 | 0.766 | 0.866 | 0.842 |
|  | N | 50 | 35 | 50 | 40 | 23 | 35 | 34 |

| Locus |  | RIN94 | RIN77 | NIS | FLY | KIL | GUD | ROS |
| --- | --- | --- | --- | --- | --- | --- | --- | --- |
| *C2-157* | Allelic richness | 11.9 | 12.5 | 13.4 | 9.1 | 12.1 | 11.1 | 12.3 |
|  | H.-W. test | 0.9461 | 0.3863 | 0.4537 | 0.0403 | 0.4747 | 0.9008 | 0.8175 |
|  | H_o_ | 1.000 | 1.000 | 1.000 | 1.000 | 1.000 | 0.971 | 0.970 |
|  | H_e_ | 0.901 | 0.911 | 0.923 | 0.854 | 0.902 | 0.890 | 0.904 |
|  | N | 50 | 36 | 50 | 40 | 24 | 35 | 33 |

^*^ significant at the 5% level, ^**^ significant at the 1% level, ^***^ significant at the 0.1% level
