# Supplementary material for: Reproductive isolation, evolutionary distinctiveness and setting conservation priorities: The case of European lake whitefish and the endangered North Sea houting (Coregonus spp.)
Source: BMC Evol Biol. 2008 May 9;8:137. doi: 10.1186/1471-2148-8-137 (PMC2396634; doi:10.1186/1471-2148-8-137)
Supplement: Additional file 2 — Historical effective population size and migration rates. Estimates of historical effective population size (θ) and migration rate between populations (M), estimated using MIGRATE 2.0.3 [58]. [file 1471-2148-8-137-S2.doc]

Supplementary Table S2. Scaled estimates of historical effective population size (*θ*; values in diagonal and denoted by bold) and migration rate between populations (*M*), estimated using MIGRATE 2.0.3.

| Immigration into | Immigration from | | | | | | |
| --- | --- | --- | --- | --- | --- | --- | --- |
|  | VID | RIN | NIS | KIL | FLY | GUD | ROS |
| VID | **0.48**  **(0.45 - 0.50)** | 10.4  (9.4 - 11.4) | 18.4  (17.1 - 19.8) | 6.6  (5.9 - 7.5) | 10.7  (9.7 - 11.8) | 12.1  (11.1 - 13.3) | 5.9  (5.2 - 6.7) |
| RIN | 17.1  (15.6 - 18.7) | **0.43**  **(0.40 - 0.47)** | 36.4  (34.2 - 38.8) | 7.2  (6.2 - 8.2) | 11.5  (10.3 - 12.9) | 13.5  (12.2 - 15.0) | 7.8  (6.7 - 8.9) |
| NIS | 26.4  (24.6 - 28.2) | 29.7  (27.9 - 31.7) | **0.53**  **(0.50 - 0.56)** | 7.7  (6.8 - 8.7) | 14.9  (13.6 - 16.3) | 16.7  (15.3 - 18.2) | 9.9  (8.8 - 1.0) |
| KIL | 15.4  (13.6 - 17.3) | 7.7  (6.4 - 9.1) | 14.4  (12.7 - 16.2) | **0.35**  **(0.32 -0.38)** | 25.0  (22.7 - 27.5) | 13.8  (12.1 - 15.6) | 4.8  (3.9 - 6.0) |
| FLY | 9.9  (8.9 - 11.0) | 11.8  (10.7 - 13.0) | 13.6  (12.4 - 14.9) | 8.9  (7.9 - 9.9) | **0.42**  **(0.39 - 0.45)** | 10.4  (9.4 - 11.6) | 8.2  (7.2 - 9.2) |
| GUD | 21.3  (19.5 - 23.2) | 12.4  (11.0 - 13.8) | 18.1  (16.4 - 19.8) | 8.1  (7.0 - 9.3) | 11.9  (10.6 - 13.4) | **0.31**  **(0.29 -0.33)** | 13.7  (12.2 - 15.2) |
| ROS | 9.2  (8.1 - 10.3) | 9.5  (8.4 - 10.6) | 13.1  (11.8 - 14.4) | 4.8  (4.0 - 5.6) | 10.4  (9.3 - 11.6) | 7.7  (6.7 - 8.7) | **0.57**  **(0.52 - 0.62)** |
